# Active Learning and the Potential of Neural Networks Accelerate Molecular Screening for the Design of a New Molecule Effective against SARS-CoV-2

**DOI:** 10.1155/2021/6696012

**Published:** 2021-05-25

**Authors:** Rabhi Yassine, Mrabet Makrem, Fnaiech Farhat

**Affiliations:** University of Tunis, The National Higher School of Engineering of Tunis (ENSIT), Laboratory of Signal Image and Energy Mastery, LR13ES03 (SIME), Tunis, Tunisia

## Abstract

A global pandemic has emerged following the appearance of the new severe acute respiratory virus whose official name is the severe acute respiratory syndrome coronavirus 2 (SARS-CoV-2), strongly affecting the health sector as well as the world economy. Indeed, following the emergence of this new virus, despite the existence of a few approved and known effective vaccines at the time of writing this original study, a sense of urgency has emerged worldwide to discover new technical tools and new drugs as soon as possible. In this context, many studies and researches are currently underway to develop new tools and therapies against SARS CoV-2 and other viruses, using different approaches. The 3-chymotrypsin (3CL) protease, which is directly involved in the cotranslational and posttranslational modifications of viral polyproteins essential for the existence and replication of the virus in the host, is one of the coronavirus target proteins that has been the subject of these extensive studies. Currently, the majority of these studies are aimed at repurposing already known and clinically approved drugs against this new virus, but this approach is not really successful. Recently, different studies have successfully demonstrated the effectiveness of artificial intelligence-based techniques to understand existing chemical spaces and generate new small molecules that are both effective and efficient. In this framework and for our study, we combined a generative recurrent neural network model with transfer learning methods and active learning-based algorithms to design novel small molecules capable of effectively inhibiting the 3CL protease in human cells. We then analyze these small molecules to find the correct binding site that matches the structure of the 3CL protease of our target virus as well as other analyses performed in this study. Based on these screening results, some molecules have achieved a good binding score close to -18 kcal/mol, which we can consider as good potential candidates for further synthesis and testing against SARS-CoV-2.

## 1. Introduction

The presence of coronaviruses constitutes a serious threat for the human population. Indeed, these viruses belong to a large family that causes a variety of diseases ranging from the common cold to more serious diseases that attack the human respiratory system. Recently, a new type of virus known as the new SARS-CoV-2 or COVID-19 coronavirus was discovered in Wuhan, China (CO-Coronavirus, VI-Coronavirus, D-December, 19-2019) [1]. So far, this new virus has caused a global pandemic [2] with more than 74,299,042 cases and 1,669,982 deaths [3] and these numbers are dangerously increasing day by day. In addition, previous outbreaks have involved severe acute respiratory syndrome viruses [4], such as “SARS-CoV” in 2003 [5] and also the Middle East respiratory syndrome virus known as “MERS-CoV” in 2012 [6]. Unfortunately, despite the existence of new vaccines that have been approved and are in use, the current lack of effective drugs against these coronaviruses has slowed the countless efforts to stop the spread of SARS-CoV-2 worldwide. And despite this delay in finding appropriate therapies, the threats posed by coronaviruses should not be underestimated and it is essential to advance our research to understand how coronaviruses replicate while interacting with their hosts so that appropriate and effective treatments can be developed as soon as possible. Furthermore, these successive outbreaks have clearly highlighted the long-term threat of interspecies transmission events leading to human epidemics and the possible reemergence of similar viral infections, which must be seriously considered [7].

Although we are familiar with this type of virus, SARS occurs suddenly, as mentioned above. This disease was quickly identified as a new coronavirus. Studies on the epidemiological, clinical and radiological characteristics of this disease showed that the infection caused severe respiratory illnesses similar to those of SARS-CoV [8]. Preliminary clinical analyses showed that although COVID-19 could cause severe disease in some patients, it was not initially easily transmissible from person to person. Nevertheless, current epidemiological data show that this novel virus is self-adapting and self-evolving in the human host and that human-to-human transmission is becoming increasingly active. Analyses performed by researchers on the SARS-CoV-2 genome sequences collected at the beginning of the epidemic have shown that they are almost identical to those of SARS-CoV [9]. Indeed, coronaviruses are members of the Coronaviridae family, which includes four genera: Alpha, Beta, Gamma, and Deltacoronavirus. Analyses show that the SARS-CoV-2 strain is a member of the Betacoronavirus family [10]. Their genomic sequence was used for genetic and functional comparison with that of the human SARS virus as well as with other coronaviruses recovered from other species. Based on phylogenetic analysis of coronaviruses from different species, the new coronavirus may have originated from bats, as its genome is about 96% identical to that of bat coronavirus, but the intermediate transmission remains to be determined [8]. As for all coronaviruses, the main structural proteins are the nucleocapsid proteins (N), membrane proteins (M), envelope proteins (E), and spike glycoprotein (S) [11, 12]. The latter plays an important role in the penetration of the virus into host cells by direct interaction with cellular receptors such as angiotensin-converting enzyme 2 (ACE2) and serine protease TMPRSS2 [13]. Upon infection, it directly triggers the process that allows the synthesis and replication of two long polyproteins [10].

Following this knowledge of the origin and genomic structure of our target, attempts are currently being made to develop small molecules capable of effectively inhibiting the main protein of SARS-CoV-2 [14]. However, current therapeutic development is focused on viral proteases. Indeed, in this area, several researchers and pharmaceutical companies are attempting to adapt existing antivirals to the novel SARS-CoV-2 protein [15]. Protease inhibitors such as remdesivir, darunavir, lopinavir, ritonavir, indinavir, saquinavir, chloroquine, and ASC-09 are in clinical trials [15]. Innovation Pharmaceuticals is evaluating brilacidin as a candidate treatment for the virus, CytoDyn is also investigating a potential treatment for the virus called leronlimab [16], and alternative approaches from the traditional Chinese medicine have also been reported [17–20]. Currently, hospital treatment relies largely on symptom-based therapies [19, 21]. Therefore, there is an urgent need to develop strategies for rapid identification of drug candidates.

**Figure 1:**
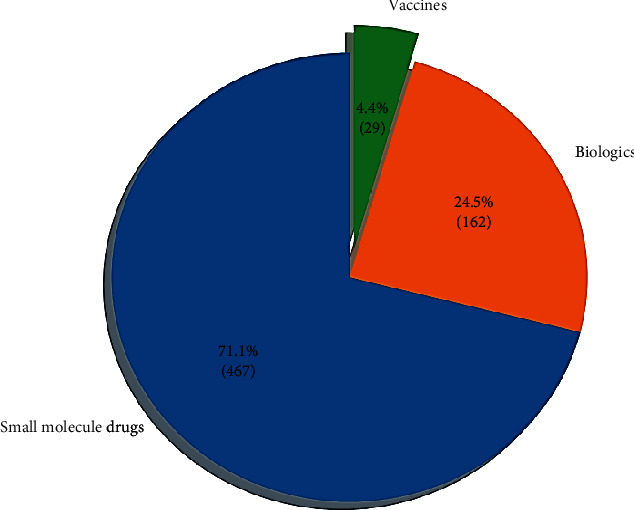
Number of trials in each therapeutic area.

Figure 1 shows the current global distribution of clinical trials against novel SARS-CoV-2 and the main types of trials [22].

However, there is still hope, especially with developments in the field of artificial intelligence (AI) that have made it possible to build on existing knowledge and use the information obtained to explore the virtually unlimited chemical space and develop new small molecules with desired biological and physicochemical properties [23–25]. Recently, AI-based methods have been used to develop new antibacterial molecules [25]. Given also that the chemical domain is too large to allow for exhaustive selection of drugs active against a specific target, techniques that allow for both design and selection of selected substances with desired properties and probability of activity are a promising approach for the future. Indeed, computer-aided design of new drugs requires extensive exploration of this vast chemical space to find compounds that may never have been synthesized before, and “deep learning” methods also provide concepts to navigate this chemical space [26]. In various research efforts, recurrent neural networks have been successfully used in activities requiring machine learning, including natural language processing [27], translation [28], and music composition [29]. One reason for this success is the technical implementation of LSTM (long-term memory), which was first used by Hochreiter and Schmidhuber in 1997 [30]. In the field of molecular informatics, LSTM-based RNNs have, among other things, been applied to predict protein functions based on their sequence [31] as well as the aqueous solubility of pharmaceutical substances [32]. In addition, AIs have been found to act as autoencoders to give a certain form of molecular structure in a chemical environment [33]. It is worth noting that several research teams have recently demonstrated the possibility of using RNNs to obtain conventional SMILES and to refine them by transfer learning [34, 35].

In this study, we used a generative and predictive model based on active learning using multiple drug databases to design novel small molecule drug-like compounds (new chemical entities known as NCEs) targeting the SARS-CoV-2 protease 3CL which is a homodimeric cysteine protease [36]. The crystal structure of the 3CL protease is available in the Protein Data Bank (PDB: 7BQY) [36]. This technique allowed us to find new molecules capable of limiting viral maturation and thus reducing infection in humans. This is done by binding all candidate drug samples and determining which one has the highest binding affinity. The drug with the highest binding strength will be a possible treatment for the target virus.

## 2. Methods

**Figure 2:**
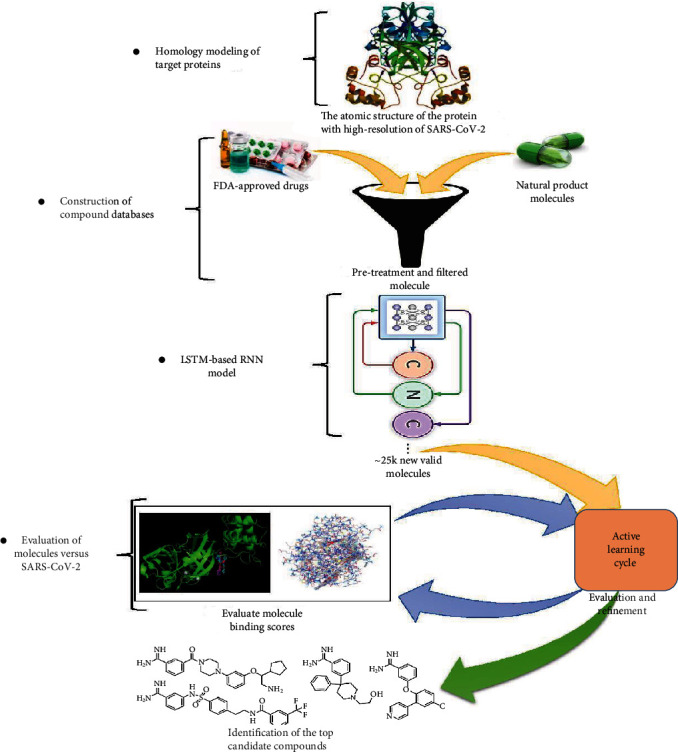
Flowchart of the strategy to identify candidate SARS-CoV-2 drugs.

Figure 2 illustrates the flow of our SARS-CoV-2 drug candidate identification strategy.

High-throughput compound screening is a time- and resource-intensive process, and considerable effort is invested in screening compound libraries, profiling, and selecting the most promising candidates for further testing. The novelty of this work, therefore, lies in the use of active learning with generative recurrent neural networks (RNNs) containing long-term memory cells (LSTMs). Active learning methods facilitate the selection process by focusing on areas of chemical space that have the best chance of success while taking into account structural novelty. The main feature of these algorithms is their ability to adapt structure-activity scenarios by feedback. To avoid performing a full screen, only targeted subsets of compounds are tested, and the experimental results are used to refine the selection of molecules in subsequent screens. Once implemented, these techniques have the potential to reduce costs and save valuable materials. The code and models are available at https://github.com/yassinerabhi/A-new-molecule-effective-against-SARS-CoV-2

### 2.1. Atomic Structure of Target Proteins

**Figure 3:**
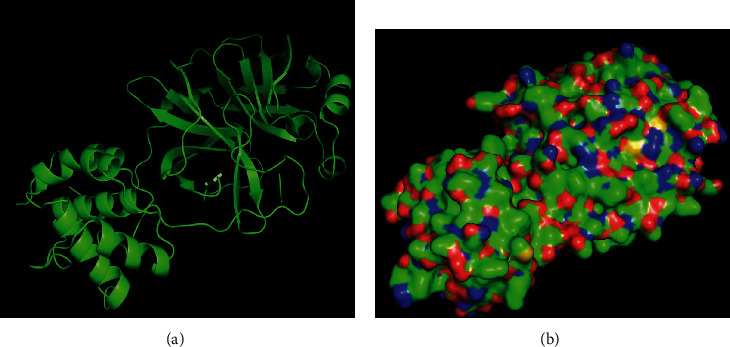
SARS-CoV-2 main protease: cartoon form (a) and surface form (b).

The high-resolution atomic structure of the SARS-CoV-2 protein (PDB ID-7BQY) was downloaded from the RCSB PDB (protein database) as previously described and processed using PyMOL, as shown in Figure 3. Prior to analysis or docking, the protein molecule was optimized using Autodock Tool 4 (ADT) [37] for molecular docking with the generated entities.

### 2.2. Construction of Compound Databases

To carry out our mission, our generative and predictive models require a large database to learn models to generate new drug molecules. Until now, the pharmaceutical industry has been responsible for much of the development and large-scale testing of molecular libraries through virtual screening. To this end, we have constructed a database that consists of (a) FDA-approved drugs (from the ZINC database), (b) natural products (from SuperNatural), and (c) a manually developed database that represents drug-like bioactive molecules. As shown in Table 1, the largest datasets correspond to the libraries used in this study were used in medicinal chemistry. Subsequently, all compounds were transformed into three-dimensional structure data files (SDF).

Our database was preprocessed and duplicates, salts, and stereochemical information were removed using “cleanup.py”, and only SMILES (simplified molecular-input line-entry system) strings between 34 and 128 in length are retained, so we get about 2492861 SMILES in total. In addition, during preprocessing, we filtered out nucleic acids and long peptides that were coming out of the chemical space we were trying to collect.

We also selected a set of drug candidates, shown in Table 2, that had been previously published with positive experimental results on coronaviruses and specifically against SARS-CoV-2. We did this to see the usefulness of these drugs and also to make a comparison with the candidate drugs that we subsequently generated.

Finally, the final list contains about 2.5 million SMILES on which the initial model was trained.

### 2.3. LSTM-Based RNN Model

In this section, we used the RNN deep learning methodology as previously described in Figure 2 to design new drugs. In the first phase of this study, we train the LSTM-based RNN model to generate reliable and high-quality SMILES. We then use transfer learning to refine the model, generating molecules that are very similar in structure to drugs with known activity against our specific SARS-CoV-2 targets.

In this way, we were able to find a generative model capable of discovering new drugs using fragment-based drug discovery (FBDD) [43] to create a library containing a series of SMILES inspired by the well-known paradigm.

To model molecules instead of language, for example with the RNN, it is enough to exchange words or letters with atoms or, more concretely, the characters of the alphabet with SMILEs, which form a formal chemical language. Indeed, if the model receives the sequence c1ccccc, there is a strong probability that the next symbol is a “1,” which closes the chemical sequence and gives benzene.

Specifically, for a sequence *S* of *S*_*i*_ symbols at steps *t*_*i*_ *ϵ* *T*, the model assigns a probability:

(1) $$\begin{array}{c} P_{\theta }\left(S\right)=P_{\theta }\left(s_{1}\right)∙\overset{T}{\underset{t=2}{\prod }}P_{\theta }\left(s_{t}\;|\;s_{t-1},\cdots ,s_{1}\right), \end{array}$$

in which the parameters *θ* are learned through the training set [44].

In this paper, we use a recurrent neural network (RNN) to estimate the probabilities associated with Equation (1). Unlike ordinary neural networks, RNNs retain state, which is essential to keep track of symbols seen previously in the chemical sequence. In general, an RNN takes a sequence of input vectors *x*_1:*n*_ = (*x*_1_, ⋯, *x*_*n*_) and an initial state vector *h*_0_, and returns a sequence of state vectors *h*_1:*n*_ = (*h*_1_, ⋯, *h*_*n*_) and a sequence of output vectors *y*_1:*n*_ = (*y*_1_, ⋯, *y*_*n*_). Finally, another function *O* allows to make the correspondence between the state vector *h*_*i*_ and the output vector *y*_*i*_ [41].

(2) $$\begin{array}{c} \text{RNN}\left(h_{0},x_{1:n}\right)=h_{1:n},y_{1:n},\\ h_{i}=R\left(h_{i-1},x_{i}\right),\\ y_{i}=O\left(h_{i}\right). \end{array}$$

Recurrent connections allow RNNs to learn complex temporal problems. In our model, RNN cells are part of the LSTM class. LSTMs have an input gate, a forget gate, an update gate, and an output gate to determine the information to be kept in a specific cell state. In this way, the hidden state of an LSTM acts as a short-term memory, while the cell state acts as a long-term memory. Therefore, LSTMs solve the problem of gradient disappearance or explosive growth that RNNs encounter due to backpropagation over long sequences.

**Figure 4:**
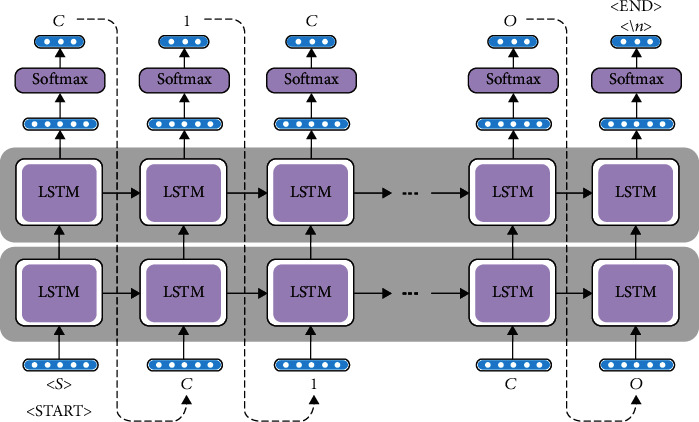
The RNN-LSTM model used to generate SMILES chains. To start, the character “*S*” is introduced, initializing the hidden and cell states. The network starts sampling symbol by symbol until the end character, “\*n*” is produced.

Figure 4 illustrates the structure of our proposed model. It consists of two LSTM layers, each having a hidden state vector of size 256, regularized by a dropout [45]. These two layers are followed by dense output layers and neurons with a Softmax activation function. Backpropagation through time was used to train the network with the cross-entropy loss function and ADAM optimizer [46, 47]. The model was created using the popular Python machine learning library TensorFlow Core v2.1.0 [48]. The input to the LSTM is a one-hot-encoded sequence of a molecule's SMILES string, where each string is split up into tokens. Each SMILES string is given an “*S*” token (for “Start”) at the beginning, and an EOL (\*n*) is added to denote the end of the SMILES string.

After training the RNN-LSTM with “Train the Network.ipynb”, the proposed model allowed us to generate about 25000 new SMILES. It is possible to generate more than this to start with a larger set of molecules to evaluate before focusing on those that react well with the SARS-CoV-2 target, but the time factor was a major constraint in this outbreak since the generation process takes several hours with our machine (laptop) whose characteristics were average.

In fact, the model was trained over 230 epochs, giving us a training accuracy of 99.86% and a validation accuracy of 99.63%. The model achieved 99.66% accuracy on a sample of test data.

And to better evaluate the relative performance of our new network, we used two parameters well known in this type of work (validity, uniqueness), and we added a third parameter (originality) to confirm the effectiveness of our method:

- Validity: out of the total number of SMILES generated, the percentages of SMILES are actually valid for the molecules
- Uniqueness: on the total number of generated valid SMILES, the percentages of SMILES are not duplicates
- Originality: out of the total number of valid SMILES generated, percentages of new creations do not appear in the training data

### 2.4. Evaluation and Refinement

When it comes to quantitative information analysis, there are many indicators and operators that we can use to identify candidate molecules. However, in addition to choosing which indicator to track, the most important thing is to define the right parameters to use. Therefore, one method we could use to find these candidate molecules without spending too much time simulating a large number of combinations would be to use active learning.

Active learning, which can also be called “selective sampling,” is a generic term in the field of machine learning for methods that select data points for testing and feeding them back into the model. Recently, this topic has gained momentum due to technological advances in small-scale organic synthesis systems and the accuracy of machine learning prediction models.

In chemical space, known activity data is provided as training data to a machine learning model that generalizes this knowledge. A selection strategy is used to choose from a list of new molecules with unknown activity. These selection strategies generally attempt to identify molecules that would be particularly suitable to improve the quality of the model (“exploratory strategies”) if they are included in the training database with their activity value. Otherwise, the molecules that could have favorable activity values are selected (“exploitation strategies”). Once the selected molecules have been tested (“tagged”), they are added to the training data to form an improved machine learning model.

Indeed, active learning is an optimization method inspired by the evolution of species and natural selection. Although it is not strictly speaking a field of machine learning, it can be a good basis for building the machine learning algorithm.

Thus, after randomly selecting 1000 SMILES and what we call “generation 0” using the “Refinement and evaluation.ipynb” script, we evaluated them with the PyRx AutoDock Vina software [49], which allowed us to obtain different scores for a diverse set of molecules. PyRx then produces a csv file of the molecules and their binding scores as well as their direct impact with the target. Subsequently, we used the techniques and principles of active learning and transfer learning to take the knowledge from the original realistic molecule creation network and transfer it to the field of creating molecules specifically capable of reacting with SARS-CoV-2.

**Figure 5:**
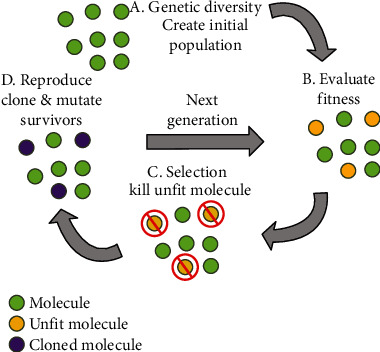
Flowchart of the strategy to identify the best binding performance candidates.

Figure 5 illustrates our technique used in this study.

For each generation that follows, we followed the following steps:

- We ordered all previously tested molecules according to their binding scores across generations and then selected the top fifty SMILES with the highest binding scores
- Next, we calculate the similarity of each remaining molecule to the set of molecules from the previous step, as well as an adjusted score that stimulates molecules that are very different from the top-ranked molecules and have good scores but not high scores, i.e., they may work by a different mechanism. Then, we take the top 10 SMILES ranked according to this adjusted similarity score
- After fundamental studies, we noticed that one of the most important characteristics of small molecules is their weight below 900 daltons [50]. We noticed that large molecules over 900 daltons seemed to have high binding affinity scores. In order to learn what made these large molecules good, but also to favor small molecules, we calculated a weight-adjusted score that favored lighter molecules with good but not great scores. We then ranked based on this adjusted score and I selected the top 10 molecules
- These steps allowed us to obtain a list of 70 molecules considered as “good fits” according to the three criteria described above: (i) global score, (ii) similarity-adjusted score (guaranteeing the inclusion of various molecules), and (iii) weight-adjusted score (guaranteeing the inclusion of particularly small molecules). In order to favor random “mutations” (inspired by a genetic algorithm approach), the RNN model already used and allowed us to generate a random sample of 10 molecules at each generation
- In total, we have 80 target SMILES (these are the “parents”). We then cumulated the results obtained by the previous generation with these 50 target SMILES. By applying a rule of thumb, we trained the network enough to minimize its loss between the first and the last epoch (5 epochs).
- Then, after retraining our model on the well-adapted “parents” of the generation, we used it to generate the next generation of ideally better-adapted “children.” In this work, we generated 500 SMILES per generation each time, which, after eliminating duplicates, invalids, etc., means that we only had a few hundred children to evaluate
- We saved the new generation in molecular SDF format and then introduced it into PyRx for evaluation

**Figure 6:**
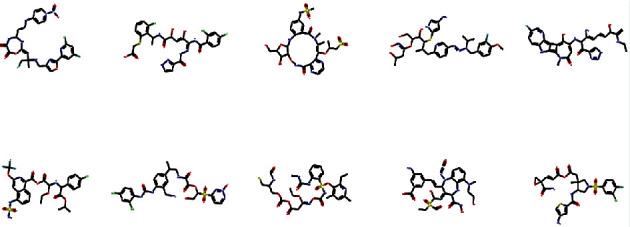
Generated SMILES molecules.

We repeated the above steps over 10 generations, always using the training set of best fit and best mutation from the previous generation to train the network to create molecules that are increasingly responsive to our target.  Figure 6 shows an example of generated molecules.

There are many software tools for virtual screening. However, the efficiency of most of these tools may not be applicable to large drug libraries such as the full list of drugs we selected from our database. In fact, we used AutoDock Vina as mentioned above as a basic docking utility to reconstruct our virtual screening pipeline and ran our smart method with all refinement processes (Figure 1) to generate the best drug candidates.

To simulate the binding affinity between protein and ligands, the 3D structure of each generated drug candidate was recorded in structure data file (SDF) format. The gen3d operation of PyRx was used for energy minimization. This operation iterated 500 cycles of geometry optimization with MMFF94 force field and weighted rotor-conformal search, to generate a probable minimum energy global conformer in MOL2 file format. Since AutoDock Vina only takes the PDBQT format as input, we used AutoDockTool to convert the file format from MOL2 to PDBQT with the default settings. After that, we applied rigid body docking on these converted files using AutoDock Vina. In order to consider all potential docking positions, the entire protein is taken as the search space in the blind search. We noted that the number of runs of the docking simulation should be adjusted accordingly considering the variety of the target protein size. In AutoDock Vina, the number of runs is defined by the completeness parameter, which was set to eight by default for a search space smaller than 30 × 30 × 30°A. We proportionally scaled the completeness to the protein size by a factor of 2. For example, if the size of a protein is 60 × 60 × 60 A, the completeness would be 8 × (60/30) × (60/30) × (60/30) × 2 = 128. AutoDock Vina showed us several docking scores for each run, and the best score was selected as the final result. Once docking is complete, AutoDock Vina generates multiple docking poses for each ligand-protein pair. To get a direct representation of the docking results, the top 20 docking poses from AutoDock Vina were taken.

## 3. Results and Discussion

In this study, two points are addressed: First, we wanted to generate a large number of diverse molecules that react and bind with high affinity. Second, we wanted to generate smaller, targeted collections enriched with molecules that are potentially active for a specific target in particular, in our case SARS-CoV-2. For the first task, we trained our model on a large general set of molecules to learn the SMILES “grammar.” This model would then allow us to generate sets of diverse but nontargeted molecules. For the second task, and in order to obtain new active molecules for our target of interest, we performed transfer learning and the principle of active learning method: We selected a small set of known active molecules for this target and retrained our pretrained chemical language model with this small dataset. After each epoch, we sampled the model to generate new actives.

Based on the results of the many experiments we conducted, we selected a model after training over 230 epochs, and the model (as described in Section 2.3) produced an average of 97.05% valid SMILES. The model was therefore selected for production runs.

**Figure 7:**
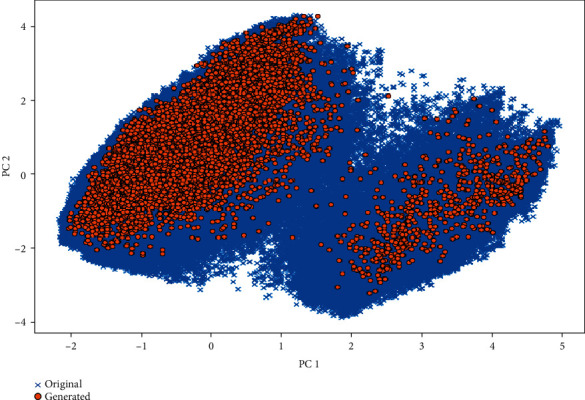
PCA projection of the molecular descriptors of the generated molecules and the original training molecules.

**Figure 8:**
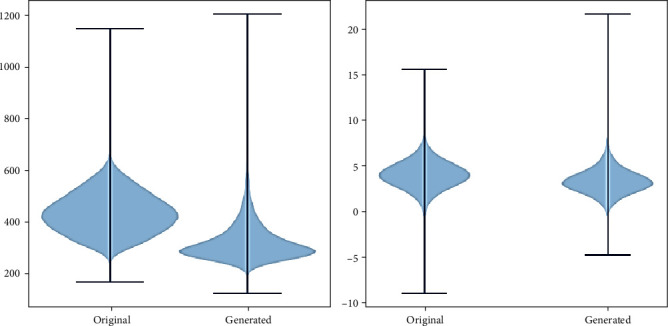
Distribution of molecular weight and calculated log*P* (clog*P*) for generated and original molecules.

Using our model, 25000 SMILES chains were generated. 99.13% of these SMILES were unique, and 94.38% of the unique SMILES were valid SMILES and 94.44% were original SMILES. In order to compare the generated molecules to the original molecules that were used for RNN training, we performed a principal component analysis (PCA) on the characteristics of the training data and the newly generated.  Figure 7 shows the original molecules as well as the generated molecules in relation to these principal components; we can see that the generated molecules were placed in the same space as the original molecules. In Figure 8, we specifically compare the molecular weight and calculated log*P* (clog*P*) distribution for the generated and original molecules. We find that the medians and distributions are very similar.

The trained generative model was used to sample 25000 small molecules from the learned chemical space as described above. After removing duplicates and identical molecules from the database used for training, the residual dataset consisted of 22173 molecules. These molecules were subjected to rigorous filters for physicochemical properties, including drug similarity [51] and synthetic accessibility [52], resulting in a set of 6962 molecules. These molecules were considered as potential candidates for the inhibition of the SARS-CoV-2 3CL protease. The generated molecules were screened for protease affinity using our evaluation and refinement method. After virtual screening, a total of 41 small molecules were obtained, with a virtual screening score of less than -7.0.

In Table 3, we took the best candidates we found in all generations (score < −7 and weight < 900 daltons) and reranked them with PyRx software, separating the highest binding score values of each molecule and its average binding score (its average over the molecule's modes in PyRx). In addition, we subsequently calculated the molecular weight since optimizing compounds for high activity on a biological target is almost always accompanied by an increase in molecular weight. However, higher weight compounds are less likely to be absorbed and thus reach the target of action. Thus, trying to keep molecular weights as low as possible should be the goal of any drug discovery, as we have already done in this study for the SARS-CoV-2 protease 3CL. In fact, more than 80% of all marketed drugs have a molecular weight below 900.

The log*P* of a compound was also calculated. It is the logarithm of its n-octanol/water partition coefficient log (*C*_octanol_/*C*_water_), a well-established measure of the compound's hydrophilicity. Low hydrophilicities and thus high log*P* values result in poor absorption or permeation. For compounds to have a good chance of being well absorbed, it has been shown that their log*P* values should not be greater than 5.0. The distribution of log*P* values calculated for many currently marketed drugs confirms this fact.

The degree of water solubility of a compound significantly affects its absorption and distribution characteristics. In general, low solubility goes hand in hand with poor absorption, so the general goal is to avoid poorly soluble compounds. The log*S* value we calculated in this work is a stripped logarithm (base 10) of the solubility measured in mol/liter. In fact, more than 80% of the drugs on the market have an (estimated) log*S* value greater than -4.

The polar surface area (PSA) was calculated in the same table; it is defined as the sum of the surface areas of all polar atoms (oxygen, nitrogen, sulfur, and phosphorus), including the fixed hydrogens. This measure is commonly used in medicinal chemistry to optimize cell permeability. Molecules with a small polar surface area, measured in square angstroms, are generally considered good for cell membrane permeability.

Finally, the similarity of each molecule was calculated with respect to existing HIV inhibitor drugs and remdesivir, which is currently in clinical trials.

As you can see, our model generated much better results than existing drugs in all tests.

**Figure 9:**
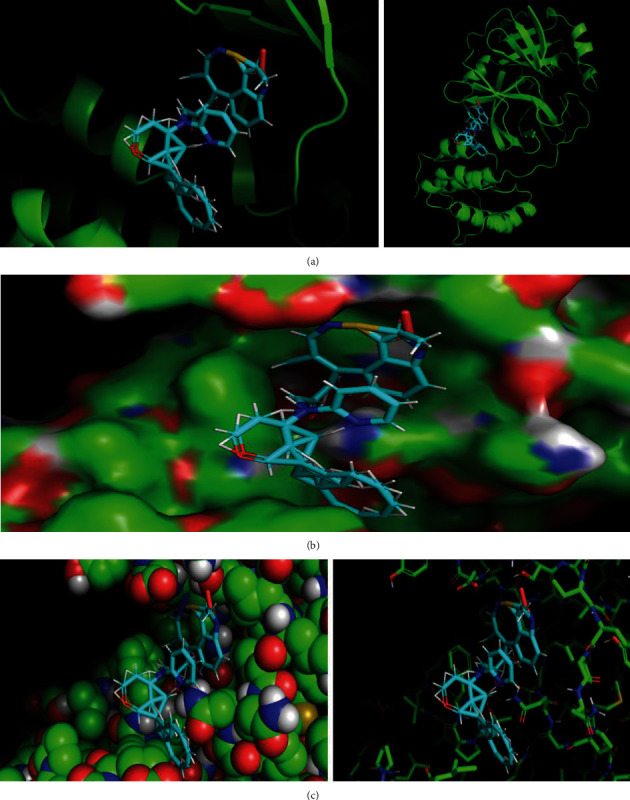
(a) The best candidate found and SARS-CoV-2 main protease (cartoon view). (b) The best candidate found and SARS-CoV-2 main protease (surface view). (c) The best candidate found and SARS-CoV-2 main protease connections.

The best docking pose for the highest compound in Table 3 is shown in Figures 9(a)–9(c). Based on comparative analysis with remdesivir and existing protease inhibitors in clinical trials, the generative model is able to accurately capture all the protease inhibitor features governing binding affinity. Indeed, these characteristics are expected to contribute to the inhibition of the SARS-CoV-2 3CL protease, resulting in a reduction of viral infection in the human body.

## 4. Conclusion

SARS CoV-2 has rapidly become a major global epidemic that has caused severe economic losses and human deaths. There is a high risk that the disease will continue to spread around the world. With a particularly high rate of transmission in the 183 affected countries and territories, it will be difficult to control this epidemic without drugs despite the existence of new vaccines that have been approved and are in use. There is an urgent need to find drugs that inhibit SARS-CoV-2.

For this purpose, small molecules were designed to inhibit the SARS-CoV-2 protease 3CL, which is responsible for viral replication. We also applied the power of advanced learning methods combined with evaluation and refinement algorithms to learn the inherent grammar of small molecules and generate new molecules that satisfy the learned grammar. Our model predicts nearly 25000 potential drugs for SARS-CoV-2 and we also used various physicochemical property filters to ensure that the generated molecules have drug-like properties. Finally, virtual screening with PyRx and AutoDock Vina was performed to obtain a ranked list of molecules. We also observed that the generative model could generate small molecules that are similar to HIV protease inhibitors, but bind better to the SARS-CoV-2 protease 3CL. A list of small molecules, which have a good virtual screening score, is also provided and presented in Table 3.

In addition, we selected the best potential anti-SARS-CoV-2 candidates for partition coefficient (log*P*), solubility (log*S*), and molecular weight (MW) analysis based on the calculated binding affinity ranking. Reasonable log*P*, log*S*, and MW show that our best anti-SARS-CoV-2 drug candidates are potentially effective in inhibiting SARS-CoV-2. Finally, the efficacy of selected anti-HIV/Ebola drugs for the treatment of SARS-CoV-2 is analyzed. Although anti-HIV drugs may indeed have a moderate effect in treating SARS-CoV-2, the analysis of these anti-HIV/Ebola drugs in combination with our best anti-SARS-CoV-2 molecules shows that the new compounds generated by our predefined method seem to have better drug properties than these HIV inhibitors.

## Figures and Tables

**Table 1:** Datasets for generation tasks.

Dataset	Purpose
ZINC [38, 39]	Commercially available compounds for virtual screening
ChEMBL [40]	A manually curated database of bioactive drug-like molecules
ChEMBL [41]	Named compounds from chemical patents
eMolecules	Purchasable molecules
Natural [42]	Natural product molecules
DrugBank	FDA-approved drugs, experimental drugs, drugs available worldwide

**Table 2:** Selected drug information of current ongoing clinical studies on SARS-CoV-2.

Drug name	Mechanism of action	Indication	DrugBank ID
Remdesivir	RNA polymerase inhibitor	Anti-Ebola passed phase III, COVID-19 phase III	DB14761
Lopinavir	Protease inhibitor	Anti-HIV approved, COVID-19	DB01601
Ritonavir	Protease inhibitor	Anti-HIV approved, COVID-19	DB00503
Emtricitabine	Nucleoside reverse transcriptase inhibitor	Anti-HIV approved, anti-HBV	DB00879
Tenofovir	Nucleoside reverse transcriptase inhibitor	Anti-HIV phase III, anti-HBV	DB14126
Ribavirin	Viral mRNA and protein synthesis inhibitor	Anti-HCV, anti-HBV, anti-SARS, anti-influenza, COVID-19	DB00811
Methylprednisolone	Corticosteroid	COVID-19 phase II, allergic asthma and rheumatic disorders approved	DB00959
Oseltamivir	Neuraminidase inhibitor; sialidase inhibitor	Anti-influenza approved, COVID-19 phase III	DB00198
Danoprevir	Protease inhibitor	Anti-HCV phase III, COVID-19 phase 4	DB11779
Chloroquine	—	Antimalarial approved, anti-HIV phase III, anti-HCV, COVID-19 phase 4	DB14761

**Table 3:** A summary of some drug properties for the top anti-SARS-CoV-2 molecules generated using our proposed method and the remdesivir and HIV drugs.

	Chemical formula (CF)	Source	Binding affinity (kcal/mol)	Molecular weight (MW)	log*P*	log*S*	PSA	Similarity to remdesivir
1	C46H50N4O8	Generated	-18.3	786.92	3.82	-6.94	190.99	0.30
2	C51H59N5O6	Generated	-18.2	838.05	4.32	-7.85	156.93	0.35
3	C52H62N6O6	Generated	-18.2	867.10	4.06	-7.62	169.82	0.38
4	C51H60N6O6	Generated	-18.1	853.07	3.72	-7.35	169.82	0.38
5	C50H58N6O6	Generated	-18	839.04	3.38	-7.08	169.82	0.38
6	C45H49N5O7	Generated	-17.7	771.91	3.20	-6.75	196.78	0.35
7	C45H48N4O8	Generated	-17.7	772.89	3.59	-6.67	190.99	0.35
8	C45H48N4O8	Generated	-17.7	772.89	3.48	-6.67	190.99	0.30
9	C52H61N5O7	Generated	-17.7	868.08	3.90	-7.72	166.16	0.42
10	C53H63N5O6	Generated	-17.7	866.11	5.01	-8.39	156.93	0.34
11	C46H52N4O7	Generated	-17.6	772.93	4.30	-7.07	173.92	0.36
12	C52H61N5O6	Generated	-17.5	852.08	4.67	-8.12	156.93	0.34
13	C52H61N5O6	Generated	-17.4	852.08	4.67	-8.12	156.93	0.34
14	C49H56N6O6	Generated	-17.3	825.01	3.03	-6.81	169.82	0.39
15	C52H66N4O9	Generated	-17.1	891.11	4.89	-8.11	211.22	0.37
16	C46H52N6O6	Generated	-16.9	784.95	3.857	-7.49	178.61	0.43
17	C44H46N4O8	Generated	-16.9	758.86	3.09	-6.25	190.99	0.40
18	C47H53N5O6	Generated	-16.7	783.96	4.80	-8.26	165.72	0.37
19	C52H62N4O6	Generated	-16.6	839.08	5.32	-8.27	148.06	0.35
20	C45H48N4O8	Generated	-16.5	772.89	3.48	-6.67	190.99	0.31
21	C45H50N6O6	Generated	-16.4	770.92	3.51	-7.22	178.61	0.43
22	C46H51N5O6	Generated	-16.2	769.93	4.46	-7.99	165.72	0.37
23	C45H48N4O8	Generated	-16.2	772.89	3.48	-6.67	190.99	0.31
24	C48H58N4O9	Generated	-16.1	835.00	3.39	-7.40	225.21	0.37
25	C53H62N6O7	Generated	-16	895.11	5.17	-9.07	194.82	0.33
26	C53H62N6O7	Generated	-16	895.11	5.30	-8.51	198.58	0.41
27	C48H58N4O10	Generated	-16	851.00	4.06	-7.12	211.66	0.46
28	C47H53N5O5	Generated	-16	767.96	4.31	-7.55	140.72	0.41
29	C44H54N6O6	Generated	-15.9	762.94	2.47	-6.49	168.96	0.37
30	C48H57N3O10	Generated	-15.8	835.99	3.79	-7.33	219.42	0.38
31	C43H51N5O6	Generated	-15.7	733.90	3.94	-7.52	165.72	0.34
32	C42H51N5O6	Generated	-15.7	721.89	4.04	-7.38	165.72	0.33
33	C43H51N5O6	Generated	-15.6	733.90	3.94	-7.52	165.72	0.34
34	C51H66N4O10	Generated	-15.5	895.10	5.06	-8.28	201.61	0.47
35	C43H50N4O6	Generated	-15.5	718.89	4.00	-7.09	153.69	0.33
36	C45H51N5O5	Generated	-15.4	741.92	3.75	-7.27	140.72	0.40
37	C43H52N4O8	Generated	-15.3	752.90	3.37	-6.34	190.99	0.31
38	C50H64N4O10	Generated	-15.1	881.07	4.76	-7.96	201.61	0.47
39	C49H62N4O11	Generated	-15	883.04	4.13	-8.09	210.84	0.46
40	C49H62N4O11	Generated	-15	883.04	4.92	-7.89	210.84	0.47
41	C49H63N5O10	Generated	-14.9	882.06	4.60	-7.62	204.85	0.52
42	C27H35N6O8P	Remdesivir	-13.2	602.58	0.30	-4.99	213.35	1.0
43	C38H53N5O7S2	HIV-TMC-310911	-11.2	755.99	5.07	-6.40	179.17	0.58
44	C38H50N6O5	HIV-saquinavir	-11.1	670.85	2.83	-5.65	166.74	0.48
45	C38H52N6O7	HIV-atazanavir	-9	704.86	3.37	-6.07	171.21	0.45
46	C27H37N3O7S	HIV-darunavir	-8.8	547.67	2.23	-3.95	148.79	0.47
47	C32H45N3O4S	HIV-nelfinavir	-8.3	567.79	4.45	-5.58	127.19	0.43
48	C25H35N3O6S	HIV-amprenavir	-8.3	505.63	2.25	-3.74	139.56	0.40
49	C36H47N5O4	HIV-indinavir	-8.1	613.80	2.84	-3.32	118.02	0.47
50	C33H44N4O6S	HIV-PPL-100	-8.1	624.80	4.18	-5.05	159.43	0.43

## Data Availability

Data are available from the authors upon request.

## Supplementary Materials

Supplementary Materials The supplementary materials contain our databases, data availability, the graphical abstract, and the final results obtained.
