# Supplementary material for: Active Learning and the Potential of Neural Networks Accelerate Molecular Screening for the Design of a New Molecule Effective against SARS-CoV-2
Source: Biomed Res Int. 2021 May 25;2021:6696012. doi: 10.1155/2021/6696012 (PMC8172298; doi:10.1155/2021/6696012)
Supplement: Supplementary Materials — The supplementary materials contain our databases, data availability, the graphical abstract, and the final results obtained. [file 6696012.f1.zip › Data Availability.docx]

**Active learning and the potential of neural networks accelerate molecular screening for the design of a new molecule effective against SARS-CoV-2**

YASSINE Rabhi *, MAKREM Mrabet, FARHAT Fnaiech

University of Tunis, The National Higher school of engineers of Tunis (ENSIT), Laboratory of Signal Image and Energy Mastery, LR13ES03 (SIME), Tunis, Tunisia.

*Correspondence : [yassinerabhi@ymail.com](mailto:yassinerabhi@ymail.com)

1. **Databases**

All compounds were prepared in (sim) data file format and the database used can be found in the attached document:

- **all_smiles.smi:** A database includes (A) FDA-approved drugs (from the ZINC database), (B) natural products (from SuperNatural) and (C) a manual database of drug-like bioactive molecules that was included in the study. All sources for the data sets were listed in Table 1 in our manuscript, with the largest data sets corresponding to the libraries used in this study and used in medicinal chemistry.

- **hiv_inhibitors_used.smi:** 14 SMILES HIV inhibitors.

- **TRPM8_inhibitors.smi:** 494 SMILES TRPM8 inhibitors.

1. **The final result obtained**

- **Final_generation_generated.sdf:** The best final results obtained are saved in an sdf file to be analyzed by the PyRx software.

- **Results_table_final.csv:** A complete table of the final results of the best candidates we found and re-classified with PyRx based on the best binding score and similarity to existing HIV inhibitor drugs and the drug Remdesivir which is currently in clinical testing.
