# Supplementary figures and images for: Active Learning and the Potential of Neural Networks Accelerate Molecular Screening for the Design of a New Molecule Effective against SARS-CoV-2
Source: Biomed Res Int. 2021 May 25;2021:6696012. doi: 10.1155/2021/6696012 (PMC8172298; doi:10.1155/2021/6696012)

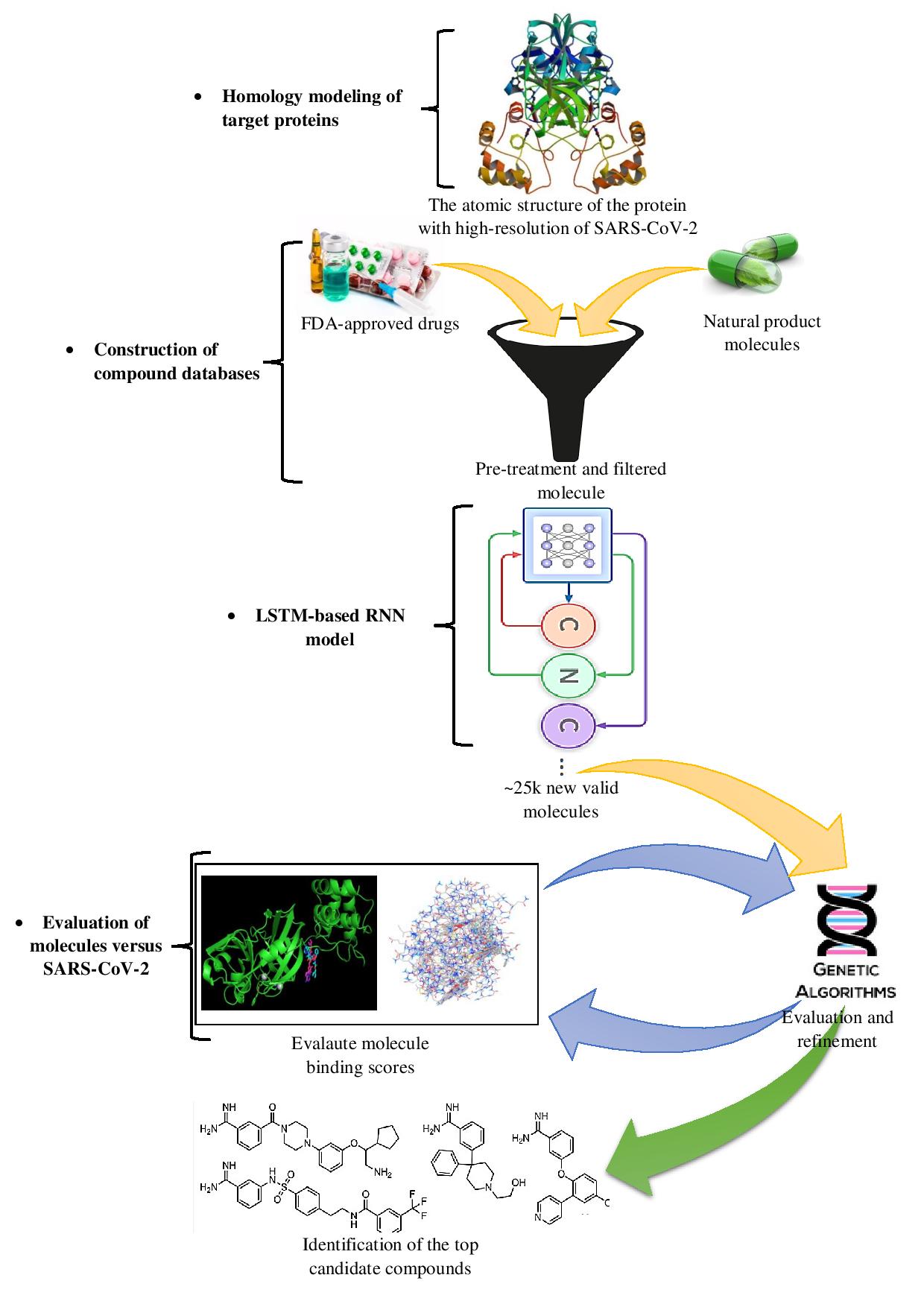


**Active learning cycle**

Supplement: Supplementary Materials — The supplementary materials contain our databases, data availability, the graphical abstract, and the final results obtained. [file 6696012.f1.zip › Graphical Abstract.docx]
